# SYVN1 ubiquitinates FoxO1 to induce β-catenin nuclear translocation, PD-L1-mediated metastasis, and immune evasion of hepatocellular carcinoma

**DOI:** 10.1007/s13402-023-00811-y

**Published:** 2023-04-26

**Authors:** Wei Xie, Lei Shi, Hu Quan, Hua Xiao, Jie Chen, Jia Liu, Jean de Dieu Habimana, Rongqi Huang, Jia Luo, Pan Chen, Zhiyuan Li

**Affiliations:** 1 Department of Anatomy and Neurobiology, School of Basic Medical Sciences, Central South University, Changsha, 410031 Hunan Province P.R. China; 2 Hunan Cancer Hospital and the Affiliated Cancer Hospital of Xiangya School of Medicine, Central South University, No. 283, Tongzipo Road, Yuelu Distirct, Changsha, 410031 Hunan Province P.R. China; 3 CAS Key Laboratory of Regenerative Biology, Guangdong Provincial Key Laboratory of Stem Cell and Regenerative Medicine, Guangzhou Institutes of Biomedicine and Health, Chinese Academy of Sciences, Guangzhou, 510530 Guangdong Province P.R. China

**Keywords:** E3 ubiquitin ligase SYVN1, FoxO1, β-catenin, PD-L1, Hepatocellular carcinoma

## Abstract

**Background:**

A high incidence of hepatocellular carcinoma (HCC), the most frequently diagnosed form of liver cancer, is observed in Africa and Asia. SYVN1 is upregulated in HCC; however, the biological roles of SYVN1 in immune evasion remain unclear.

**Methods:**

RT-qPCR and western blot were employed to detect the expression levels of SYVN1 and the key molecules in HCC cells and tissues. Flow cytometry was used to determine the proportion of T cells, and an ELISA assay was used to determine the amount of IFN-γ secreted. Cell viability was monitored by CCK-8 and colony formation assays. The metastatic properties of HCC cells were detected by Transwell assays. Bioinformatics analysis, ChIP, and luciferase assays were used to study the transcriptional regulation of PD-L1. Co-IP was used to detect direct interaction between SYVN1 and FoxO1, as well as the ubiquitination of FoxO1. The in vitro findings were validated in xenograft and lung metastasis models.

**Results:**

In HCC cells and tissues, SYVN1 was upregulated while FoxO1 was downregulated. SYVN1 knockdown or FoxO1 overexpression reduced PD-L1 expression, and inhibited immune evasion, cell growth, and metastasis in HCC cells. Mechanistically, FoxO1 regulated PD-L1 transcription in a β-catenin-independent or -dependent manner. Functional studies further showed that SYVN1 promoted immune evasion, cell proliferation, migration and invasion via facilitating ubiquitin-proteasome-dependent degradation of FoxO1. In vivo investigations showed that silencing of SYVN1 inhibited immune evasion and metastasis of HCC cells, possible via the FoxO1/PD-L1 axis.

**Conclusion:**

SYVN1 regulates FoxO1 ubiquitination to stimulate β-catenin nuclear translocation and promotes PD-L1-mediated metastasis and immune evasion in HCC.

**Supplementary Information:**

The online version contains supplementary material available at 10.1007/s13402-023-00811-y.

## Introduction

Liver cancer is the sixth most common malignancy with high mortality globally [1]. Hepatocellular carcinoma (HCC) accounts for ~ 90% of liver cancers [2]. HCC is most frequent in Africa and Asia, with Asia accounting for 75% of all new cases and deaths [3, 4]. Currently, surgical resection and liver transplantation remain the curative approaches for HCC. Unfortunately, the majority of HCC patients cannot benefit from these curative treatments due disease progression or organ shortage. Metastasis remains the major challenge for HCC treatment [5]. Emerging research has focused on immune evasion as a key event during tumor metastasis [6]. In recent years, novel therapeutic strategies for advanced or metastatic HCC, such as immune checkpoint inhibitors (ICIs), types of monoclonal antibody drugs, have been approved [2, 7, 8]. Therefore, unraveling the molecular mechanism of the novel agents, particularly ICIs, could provide important insights into clinical practice. Cancer cells can escape immune surveillance, leading to cancer initiation and metastasis [9].Different mechanisms of immune escape have been illustrated, such as genetic alterations in cancer cells and perturbations in the tumor-immune microenvironment [9]. The association between programmed cell death 1 (PD-1) on T cells and its ligand PD-L1 on tumor cells suppresses activation, growth, cytotoxic secretion, and survival of T cells, resulting in cancer immune evasion [10, 11].Previous studies have associated PD-L1 overexpression with aggressiveness, poor prognosis, and recurrence in HCC [12, 13]. Currently, the ICIs, such as anti-PD1 and anti-CTLA-4 antibodies, exhibit promising results in patients with advanced HCC; however, anti-PD-L1 antibodies are still under evaluation [14, 15]. Additionally, the underlying mechanism by which PD-L1 is upregulated in HCC cells remains ambiguous.

Synoviolin (SYVN1) is an endoplasmic reticulum (ER)-resident E3 ubiquitin ligase, and it is implicated in the tumorigenesis of various cancers by modulating the ubiquitin-proteasome-dependent degradation of key molecules, such as p53 and Sirtuin 2 [16, 17]. More importantly, the ubiquitinomic analysis revealed that SYVN1 is overexpressed in HCC and contributes to tumorigenesis and metastasis [18]. In addition, FoxO1 is known to have reduced expression and to function as a tumor suppressor in HCC [19]. FoxO1 interacts with β-catenin in the cytosol and restrains β-catenin from being enriched in the nucleus in HCC cells, thus suppressing β-catenin-mediated transcriptional regulation of target genes [20]. Intriguingly, previous research has shown that β-catenin transcriptionally induces PD-L1 expression to promote immune escape in glioblastoma [21, 22]. However, it remains unclear whether FoxO1/β-catenin serves as an upstream regulatory signaling of PD-L1 in HCC. Bioinformatics analysis through Ubibrowser (http://ubibrowser.bio-it.cn/ubibrowser/) predicted that SYVN1 mediated ubiquitination and degradation of FoxO1, and putative binding sites of FoxO1 on the PD-L1 promoter were also predicted by AnimalTFDB (http://bioinfo.life.hust.edu.cn/AnimalTFDB/#!/). Thus, we hypothesized that SYVN1 could mediate ubiquitin-proteasome-dependent degradation of FoxO1. On one hand, we postulated that downregulated FoxO1 may stimulate β-catenin nuclear translocation, promoting β-catenin-mediated transcriptional activation of PD-L1. Additionally, FoxO1 may act as a transcriptional suppressor of PD-L1 by directly interacting with the PD-L1 promoter. The upregulation of PD-L1 could further contribute to HCC immune evasion and metastasis. In this study, we found that in HCC cells and tissues, FoxO1 was downregulated whereas SYVN1 was upregulated. FoxO1 overexpression or SYVN1 knockdown decreased PD-L1 expression and inhibited immune evasion, metastasis, and cell proliferation in HCC cells. FoxO1 regulated the expression of PD-L1 either independently or dependently of β-catenin. SYVN1 promoted immune escape, cell proliferation, and metastasis via regulating ubiquitin-proteasome-dependent degradation of FoxO1. Our in vivo findings further showed that SYVN1 silencing inhibited immune evasion and metastasis of HCC cells, possibly through the FoxO1/PD-L1 axis.

## Materials and methods

### Clinical specimens

A cohort of 30 HCC tissues and their normal counterparts were collected from HCC patients from Hunan Cancer Hospital and the Affiliated Cancer Hospital of Xiangya School of Medicine, Central South University. Written consents from all patients were obtained. This study was approved by the Ethics Committee of Hunan Cancer Hospital and the Affiliated Cancer Hospital of Xiangya School of Medicine, Central South University.

### Cell culture, treatment and transfection

Normal human liver cell line THLE-3 (RRID: CVCL_3804), HCC cell lines Hep3B (RRID: CVCL_0326), and HEK293T cells (RRID: CVCL_0063) were purchased from ATCC (Manassas, VA, USA). Li-7 (RRID: CVCL_3840), HuH-7 (RRID: CVCL_0336), SNU-182(RRID: CVCL_0090), and HuH-6 (RRID: CVCL_4381) cells were provided by Cell Bank/Stem Cell Bank, Chinese Academy of Sciences. MHCC97H (RRID: CVCL_4972) and BEL-7405 (RRID: CVCL_6569) cells were purchased from Fenghbio (Changsha, China). These cell lines are not listed as a commonly misidentified cell line by the ICLAC. In this study, THLE-3 and all HCC cells with less than 15 generations were used. The cells were grown in RPMI 1640 and DMEM containing 10% FBS (Gibco, Grand Island, NY, USA), respectively. All cells were cultured at 37 °C with 5% CO_2_, and all experiments were performed with mycoplasma-free cells. To study the protein stability and degradation, cells were treated with 20 µg/mL of CHX or MG132 for 24 h. sh-NC, sh-SYVN1-1, sh-SYVN1-2, sh-β-catenin-1 or sh-β-catenin-2 were from GenePharma (Shanghai, China). The full-length of FoxO1 or SYVN1 was cloned into the pcDNA3.1 vector. SFB-SYVN1, SFB-FoxO1, Myc-FoxO1, and HA-Ubiquitin were constructed as previously described [23]. HCC cells were transfected using Lipofectamine 3000 (Invitrogen, Carlsbad, CA, USA).

### PBMCs isolation and co-culture of HCC cells with PBMCs

PBMCs were isolated from peripheral blood of healthy donors using a Ficoll procedure as previously described [24]. PBMCs were grown in RPMI 1640 containing 10% FBS (Gibco) overnight. To activate T cells, the suspended cells were harvested and stimulated with 10 µg/mL PHA and 4000 UI/mL rhIL-2 for 48 h. T cells were then cultured in RPMI 1640 containing 2000 UI/mL rhIL-2 and 10% FBS as previously described [25]. For co-culture, HCC cells were plated into a 24-well plate and cultured in complete DMEM. PBMCs were placed in culture inserts (Corning, NY, USA) at 5 × 10^5^ cells/well as previously described [26].

### RT-qPCR

Total RNA was extracted from tissues and cells using Trizol reagent (Invitrogen). cDNA was synthesized using the Advantage RT-PCR Kit (TaKaRa, Dalian, China), and RT-qPCR was carried out using the iQ SYBR Green Supermix (Bio-Rad, Hercules, CA, USA). The target gene’s levelwas determined using 2^–ΔΔCT^ method. The primers used in RT-qPCR are listed in Supplementary Table 1.

### Western blot

Protein lysates were extracted using RIPA lysis buffer (Pierce, Rockford, IL, USA). Protein concentration was estimated using Bradford assay (Bio-Rad, Hercules, CA, USA). Proteins were resolved in SDS-PAGE and transferred to a PVDF membrane (Bio-Rad). After blocking, the blot was incubated with the primary antibody. This was followed by the incubation with a corresponding secondary antibody. The signal was detected using the ECL detection system (Pierce, city, country). Antibodies used in western blot were listed in Supplementary Table 2.

### Immunofluorescence (IF)

HCC cells were fixed and permeabilized. After blocking with 1% BSA, the slides were incubated with anti-PD-L1 antibody (1:200, ab205921, Abcam) at 4 °C overnight. Cells were then incubated with Alexa Fluor 488-conjugated secondary antibody (Invitrogen) and mounted with Prolong Gold with DAPI mounting medium (Invitrogen). Images were acquired by a Nikon confocal microscope (Nikon, Tokyo, Japan).

### Flow cytometry

The percentages of CD4 + and CD8 + T cells in the co-culture system of HCC cells and PBMCs were assessed by flow cytometry. After staining with anti-CD3, anti-CD4 FITC, or anti-CD8 FITC (Invitrogen), data were acquired using a FACSCalibur flow cytometer (BD Biosciences, Franklin Lakes, NJ, USA).

### ELISA assay

A human IFN- ELISA kit (Abcam, Cambridge, MA, UK) was used to measure the amount of secreted IFN-γ. In brief, the collected culture medium was centrifuged at 1400 rpm for 1 min. The ELISA assay was conducted according to the manufacturer’s protocols, and A450 was measured using a microplate reader (Bio-Rad).

### Cell counting Kit-8 (CCK-8) assay

HCC cells (3 × 10^3^) were seeded into 96-well plates. At the designated time points, 20 µL (per well) of CCK-8 solution (Beyotime, Jiangsu, China) was added and incubated at 37 °C for 1 h. A450 was detected using a microplate reader (Bio-Rad).

### Colony formation assay

HCC cells (5 × 10^2^ cells/well) were plated into a 6-well plate. After 2 weeks, cells were fixed and stained with crystal violet. The stained colonies were photographed and counted.

### Transwell assay

Transfected HCC cells (1 × 10^3^) were seeded in the upper chamber (Corning) and cultured in serum-free DMEM. The lower chamber was filled with complete DMEM. The migrated cells were fixed and stained with crystal violet after 24 h. The invasion assay was conducted using a similar approach with the Matrigel (Corning) coating.

### Chromatin immunoprecipitation (ChIP) assay

The ChIP assay was carried out using the EZ-ChIP Kit (Millipore, Billerica, MA, USA). Hep3B and MHCC97H cells were crosslinked and lysed, and the lysates were subjected to sonication. After that, the chromatin fractions were then incubated with anti-β-catenin (1 µg, ab32572, Abcam), anti-FoxO1 antibody (1 µg, ab39670, Abcam) or normal rabbit IgG. The purified DNA was analyzed by RT-qPCR.

### Dual-luciferase reporter assay

A series of luciferase plasmids containing the PD-L1 promoter were generated and cloned into pGL-3 (Promega, Madison, WI, USA). The mutated construct was generated using the QuikChange mutagenesis kit (Agilent, Santa Clara, CA, USA). FoxO1 overexpression plasmid/sh-β-catenin and luciferase construct were co-transfected into HCC cells. Luciferase activity was assessed using Dual luciferase system (Promega, Madison, WI, USA).

### Co-immunoprecipitation (Co-IP)

The full-length of SYVN1 or FoxO1 was constructed into the pMH-SFB vector. SFB-SYVN1 was made up of an S-peptide, a Flag peptide, and streptavidin-binding peptide. The enrichment of SFB-SYVN1 was performed using streptavidin beads (Pierce) as previously described [23]. For co-IP, cell lysates were incubated with anti-SYVN1 antibody (1 µg, ab170901, Abcam) or normal rabbit IgG. The protein complex was then immunoprecipitated using protein A/G beads (Pierce). The eluted proteins were analyzed by western blot.

### Animal study

The animal study was approved by the Ethics Committee of Hunan Cancer Hospital and the Affiliated Cancer Hospital of Xiangya School of Medicine, Central South University. NOD/SCID mice were from the Shanghai Experimental Animal Center of the Chinese Academy of Sciences (Shanghai, China). For xenograft experiment, NOD/SCID mice were implanted with 2 × 10^6^ HCC cells stably transfected with shNC or sh-SYVN1-1 and sh-SYVN1-2 through subcutaneous injection. When the tumor volume was more than 100 mm^3^, the mice were administered with human immunocyte mixtures which consisted of PBMCs (5 × 10^6^ /per mouse) and activated T lymphocytes (1 × 10^7^ /per mouse) or PBS via tail intravenous injection once a week for three times. After post-implantation, all mice were sacrificed, and the tumor inhibition rate was calculated. Tumors were measured every 7 days, and the tumor volume was calculated (1/2 × length ×width^2^). Tumor weight was measured on day 28 post-inoculation.

### Histological analysis

The xenograft tumor or lungs were dissected, fixed, and paraffin-embedded. The sections were stained with H&E [27]. For immunohistochemistry (IHC) analysis, the slides were stained with anti-PD-L1 (1:200, ab205921, Abcam), Ki-67 (1:200, ab15580, Abcam), SYVN1 (1:100, ab170901, Abcam), and FoxO1 (1:100, ab39670, Abcam) at 4 °C overnight. The slides were then incubated with secondary antibody. The signal was detected using the DAB substrate (Beyotime).

### Statistical analysis

Data were analyzed with GraphPad Prism 8.0 (San Diego, CA, USA) and presented as mean ± S.D. One-way ANOVA or Student’s *t*-test was conducted to assess the differences. *P* < 0.05 was considered statistically significant.

## Results

### SYVN1 overexpression downregulates FoxO1 in HCC cells and tissues

**Fig. 1:**
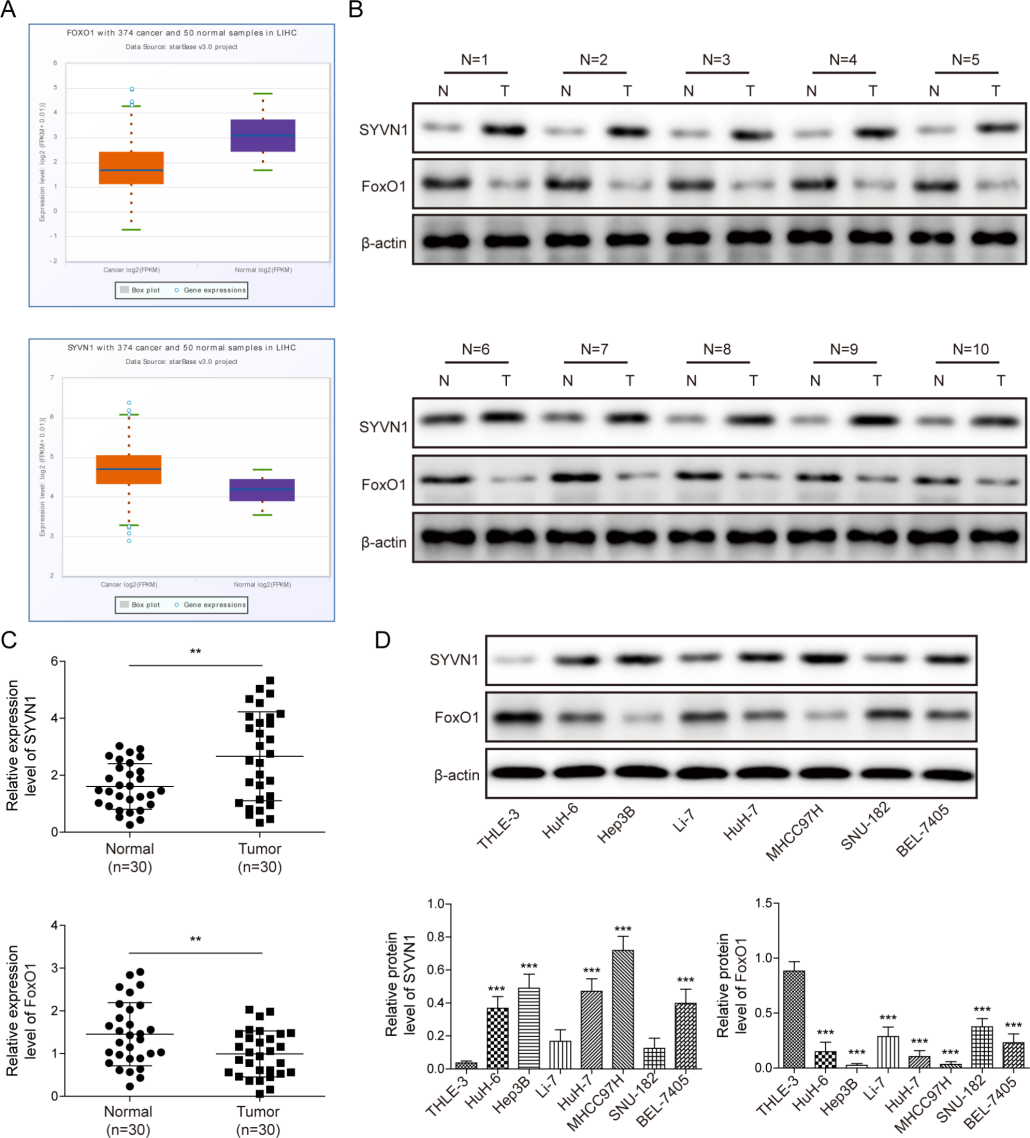
SYVN1 is upregulated, and FoxO1 is downregulated in HCC tissues and cells. (**A**) Data analyses based on TCGA database. (**B**) The protein levels of SYVN1 and FoxO1 in HCC tissues (n = 10) were detected by western blot. (**C**) The mRNA levels of SYVN1 and FoxO1 in HCC tissues (n = 30) were detected by RT-qPCR. (**D**) The protein levels of SYVN1 and FoxO1 in HCC cells were detected by western blot. **, *P* < 0.01, ***, *P* < 0.001

According to bioinformatics analysis (http://ubibrowser.bio-it.cn/ubibrowser/home/index), SYVN1 may be involved in the ubiquitination and degradation of FoxO1.To test this regulatory mechanism, the expression of SYVN1 and FoxO1 in HCC were examined. Analysis from TCGA data revealed that SYVN1 was significantly increased in HCC tissues; however, FoxO1 was significantly decreased (Fig. 1A). Western blot analysis consistently revealed that SYVN1 or FoxO1 was induced or reduced in HCC tissues (n = 10) when compared to their normal counterparts (Fig. 1B). These findings were further confirmed by RT-qPCR (n = 30) (Fig. 1C). We next examined the levels of SYVN1 and FoxO1 in different HCC cell lines. In comparison with normal human liver cell line, THLE-3 cells, SYVN1 was highly expressed while FoxO1 was poorly expressed in all seven HCC cells, including HuH-6, Hep3B, Li-7, HuH-7, MHCC97H, SNU-182 and BEL-7405 cells (Fig. 1D). Additionally, clinical data analysis revealed that increased SYVN1 levels were associated with larger tumor sizes, lower blood NK cell proportions, and greater microvascular invasion (Supplementary Table 3). These findings demonstrate that FoxO1 is downregulated in HCC as a result of SYVN1 overexpression.

### SYVN1 silencing decreases PD-L1 expression, and inhibits immune escape, cell growth, and metastasis in HCC cells

**Fig. 2:**
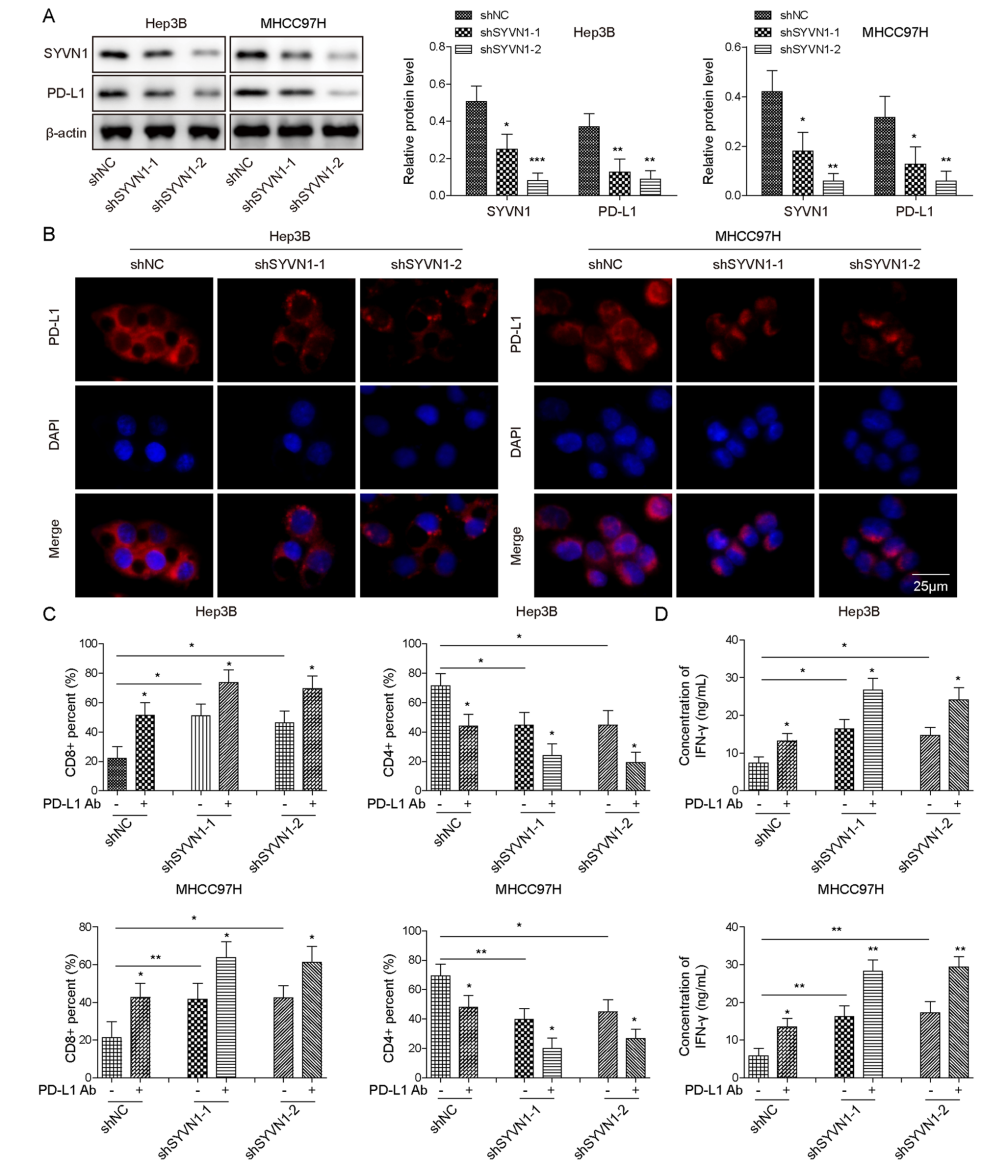
Knockdown of SYVN1 decreases PD-L1 expression, and inhibits immune escape in HCC cells. (**A**) The protein levels of SYVN1 and PD-L1 in Hep3B and MHCC97H cells were detected by western blot. (**B**) Immunofluorescence staining of PD-L1 in Hep3B and MHCC97H cells were detected. Red, PD-L1; Blue, DAPI. Scale bar, 25 μm. (**C**) The proportion of CD3+/CD8 + or CD3+/CD4 + T cells were detected by flow cytometry. (**D**) The secreted IFN-γ level was assessed by ELISA assay. *, *P* < 0.05, **, *P* < 0.01

To investigate the biological role of SYVN1 in immune evasion, cell growth, and metastasis, knockdown studies were carried out in HCC cells. To delineate the biological function of SYVN1 in HCC, knockdown experiments were conducted in Hep3B and MHCC97H cells with relatively high expression of SYVN1. As presented in Fig. 2A, SYVN1 and PD-L1 expression were both downregulated in HCC cells after transfection with sh-SYVN1-1 or sh-SYVN1-2. In agreement with these data, IF showed that PD-L1 staining intensity was markedly decreased in SYVN1-knockdown cells (Fig. 2B). In the HCC and PBMCs co-culture model, SYVN1 knockdown or PD-L1 Ab increased the percentage of CD3+/CD8 + T cells while decreasing the proportion of CD3+/CD4 + T cells. A more prominent change in the proportion of CD8 + T or CD4 + T cells was observed in the sh-SYVN1 + PD-L1 Ab group (Fig. 2C). Moreover, the culture supernatant showed a remarkable induction of IFN- γ in response to the silencing of SYVN1 or PD-L1 Ab, and the secreted IFN-γ levels in the sh-SYVN1 + PD-L1 Ab group were much greater than in the corresponding control group (Fig. 2D). Furthermore, the CCK-8 and colony formation assays revealed that SYVN1 knockdown suppressed cell proliferation and colony formation in both Hep3B and MHCC97H cells (Supplementary Fig. 1A-B). Transwell assays unequivocally showed that the lack of SYVN1 impaired the migratory and invasive capacities of HCC cells (Supplementary Fig. 1C-D). These findings suggest that SYVN1silencing inhibits immune escape, cell growth, and metastasis in HCC cells by reducing PD-L1 expression.

### FoxO1 overexpression decreases PD-L1 level, and suppresses immune escape, cell growth, and metastasis in HCC cells

**Fig. 3:**
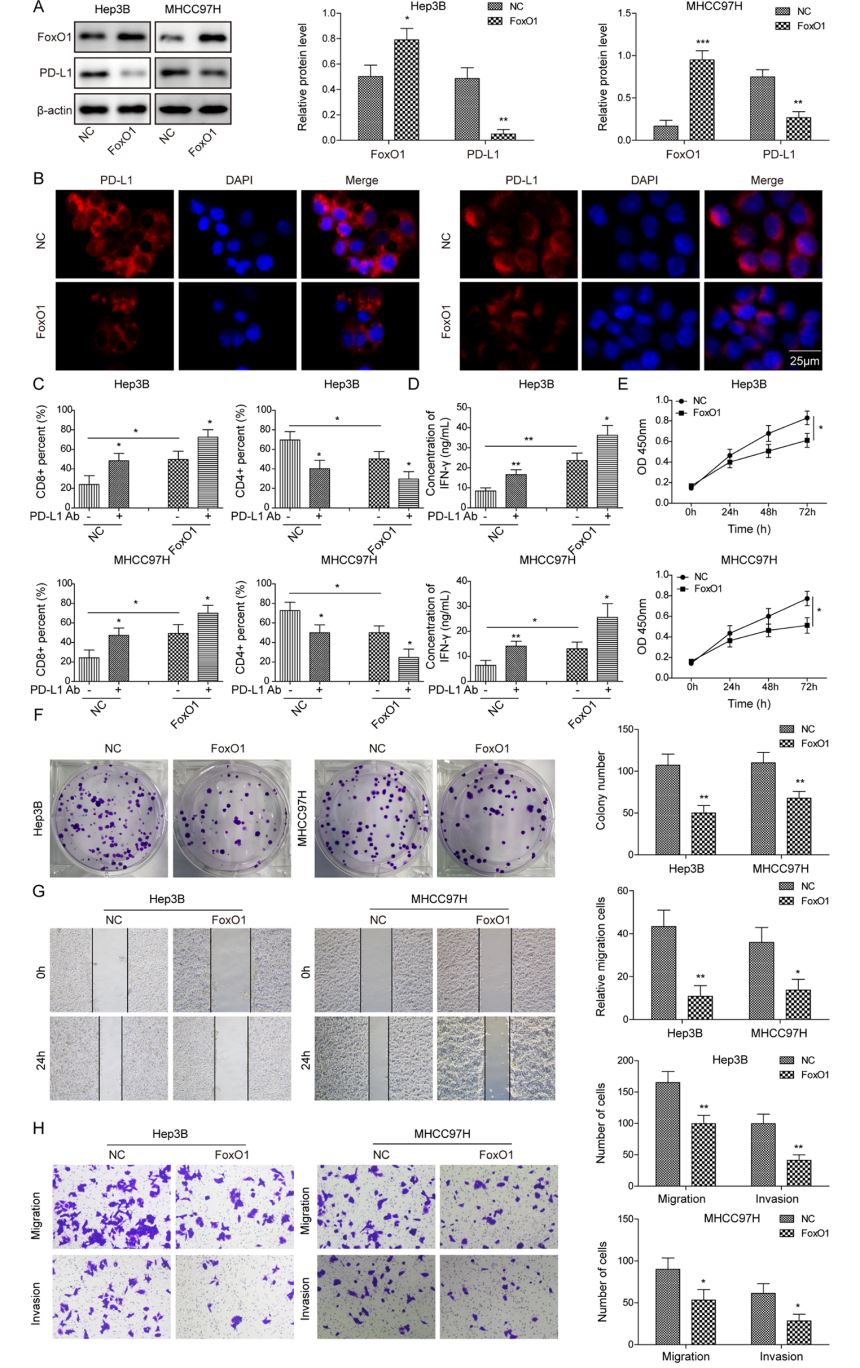
FoxO1 overexpression decreases PD-L1 level, and suppresses immune escape, cell proliferation and metastasis in HCC cells. (**A**) The protein levels of FoxO1 and PD-L1 in Hep3B and MHCC97H cells were detected by western blot. (**B**) Immunofluorescence staining of PD-L1 in Hep3B and MHCC97H cells were detected. Red, PD-L1; Blue, DAPI. Scale bar, 25 μm. (**C**) The proportion of CD3+/CD8 + or CD3+/CD4 + T cells were detected by flow cytometry. (**D**) The secreted IFN-γ level was assessed by ELISA assay. (**E**) Cell proliferation was monitored by CCK-8 assay. (**F**) Colony forming ability was assessed by colony formation assay. (**G**) Cell migration and invasion were detected by Transwell assay. *, *P* < 0.05, **, *P* < 0.01, ***, *P* < 0.001

To further investigate the role of FoxO1 in HCC, we performed overexpression experiments. As expected, transfection of the FoxO1 overexpression construct resulted in a significant increase in FoxO1 and a significant decrease in PD-L1 in both Hep3B and MHCC97H cells (Fig. 3A). Similarly, the staining intensity of PD-L1 was decreased in FoxO1-overexpressing Hep3B and MHCC97H cells (Fig. 3B). Flow cytometry revealed that FoxO1 overexpression or PD-L1 Ab increased CD3+/CD8 + T cells while decreasing CD3+/CD4 + T cells. FoxO1 + PD-L1 Ab resulted in a more prominent change in the proportion of CD8 + T or CD4 + T cells (Fig. 3C). In accordance with these findings, the ELISA assay further revealed IFN-γ induction in FoxO1 overexpression, PD-L1 Ab, and FoxO1 + PD-L1 Ab groups. It is worth noting that the level of secreted IFN-γ was significantly higher in FoxO1 + PD-L1 Ab groups than in FoxO1 overexpression or PD-L1 Ab alone groups (Fig. 3D). Interestingly, FoxO1overexpression inhibited cell growth and colony formation in HCC cells (Fig. 3E-F). Additionally, the metastatic properties of HCC cells were impaired by FoxO1 overexpression (Fig. 3G). These data indicate that FoxO1 overexpression and SYVN1 knockdown exert similar inhibitory effects on PD-L1 expression, immune escape, cell growth, and metastasis in HCC cells.

### FoxO1 regulates PD-L1 level in a β-catenin-independent or -dependent manner

**Fig. 4:**
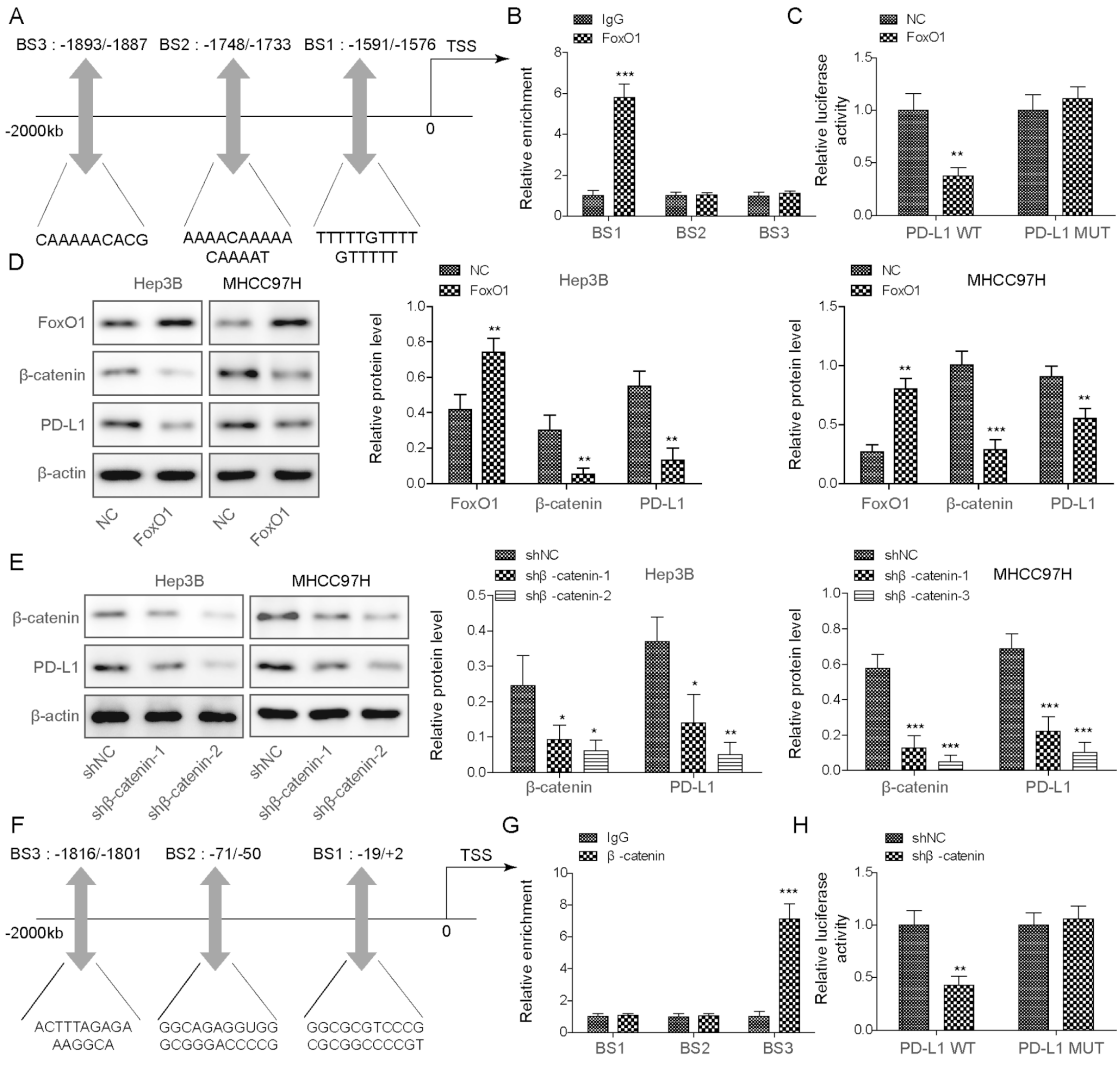
FoxO1 regulates PD-L1 expression in a β-catenin-independent or -dependent manner. (**A**) The putative binding sites between FoxO1 and PD-L1 promoter were predicted by AnimalTFDB. (**B**) The enrichment of FoxO1 on PD-L1 promoter was assessed by ChIP assay. (**C**) The relative luciferase activity was detected by dual-luciferase reporter assay. (**D**) The protein levels of FoxO1, β-catenin and PD-L1 were detected by western blot. (**E**) The protein levels of β-catenin and PD-L1 were detected by western blot. (**F**) The putative binding sites between β-catenin and PD-L1 promoter were predicted by AnimalTFDB. (**G**) The enrichment of β-catenin on PD-L1 promoter was assessed by ChIP assay. (**H**) The relative luciferase activity was detected by dual-luciferase reporter assay. *, *P* < 0.05, **, *P* < 0.01, ***, *P* < 0.001

FoxO1 was predicted as a transcription factor of PD-L1 by the Animal TFDB database. The putative binding sites of FoxO1 on the PD-L1 promoter, namely, BS1 (-1591/-1576), BS2 (-1748/-1733), and BS3 (-1893/-1887), were shown in Fig. 4A. The ChIP assay showed a significant enrichment of FoxO1 at the BS1 (-1591/-1576) promoter region (Fig. 4B), suggesting that FoxO1 is preferably bound to the BS1 region of the PD-L1 promoter. The wild-type (PD-L1 WT) or BS1 region mutated PD-L1 promoter (PD-L1 MUT) was further constructed into a pGL-3 vector and co-transfected with the vector alone or the FoxO1 overexpression construct. The Luciferase assay revealed that co-transfection of PD-L1 WT and FoxO1 resulted in a significant reduction of promoter activity, which was abolished by PD-L1 MUT (Fig. 4C), indicating a direct association between FoxO1 and the PD-L1 promoter. Intriguingly, western blot showed that FoxO1overexpression decreased β-catenin and PD-L1 expression in both Hep3B and MHCC97H cells (Fig. 4D). We next sought to test whether β-catenin was involved in the transcriptional regulation of PD-L1. A knockdown study revealed that the lack of β-catenin led to a reduced expression of PD-L1 in both Hep3B and MHCC97H cells (Fig. 4E). Based on Animal TFDB, three putative binding sites between β-catenin and PD-L1 promoter were predicted, including BS1 (-19/+2), BS2 (-71/-50) and BS3 (-1816/-1801) (Fig. 4F). Among these putative binding sites, BS3 (-1816/-1801) was responsible for β-catenin interaction as determined by the ChIP assay (Fig. 4G). β-catenin depletion significantly decreased the activity of the PD-L1 WT but not the BS3-mutated (PD-L1 MUT) promoter (Fig. 4H), indicating that β-catenin acts as a transcription activator of PD-L1. These findings suggest that FoxO1 regulates PD-L1 level in a β-catenin-independent or -dependent manner.

### SYVN1 mediates FoxO1 degradation via ubiquitin-proteasome pathway

**Fig. 5:**
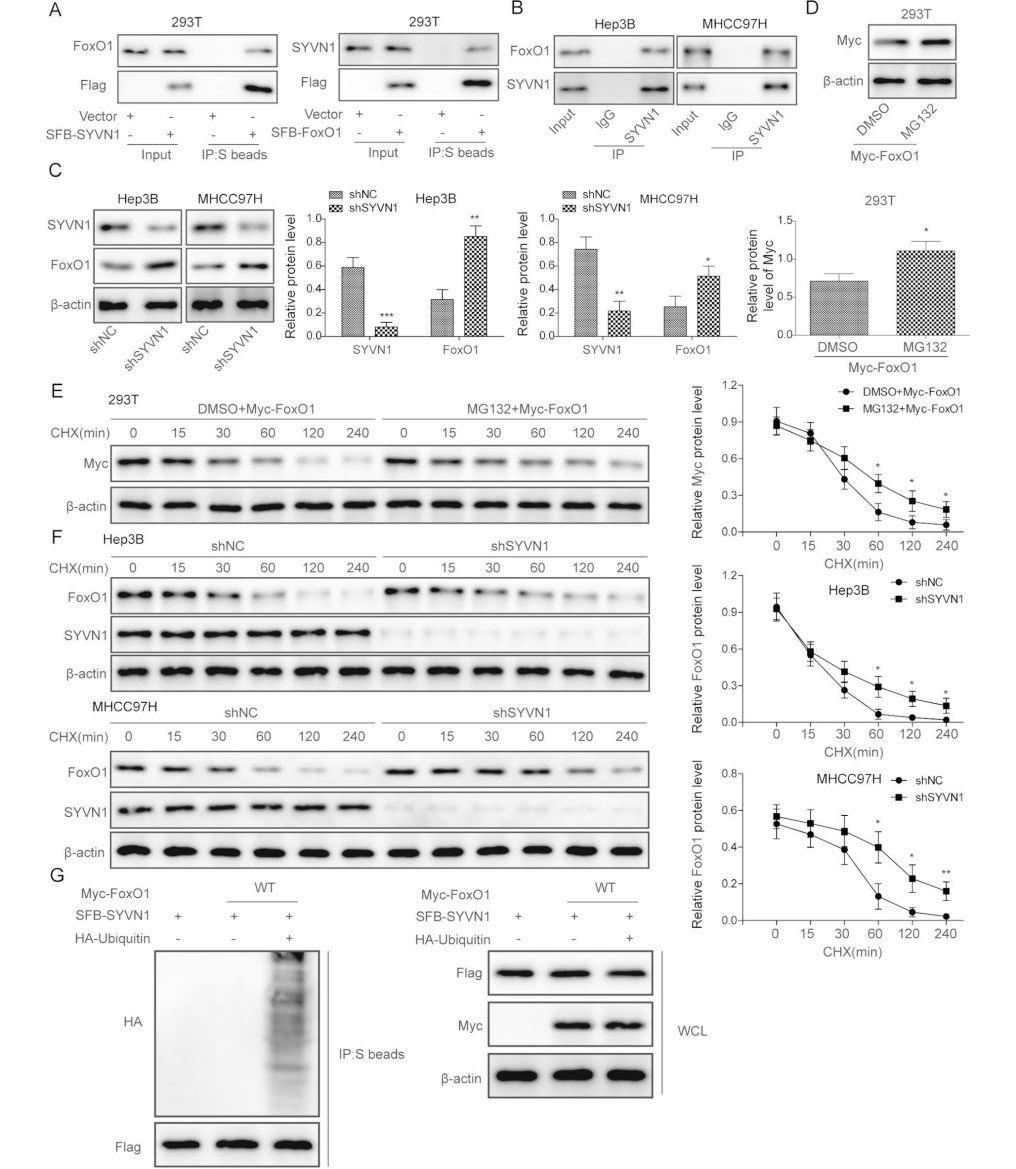
SYVN1 mediates FoxO1 degradation via ubiquitin-proteasome pathway. (**A**) S beads-enriched complex was detected by western blot. (**B**) The direct interaction between SYVN1 and FoxO1 in Hep3B and MHCC97H cells was assessed by Co-IP. Normal IgG served as a negative control. Whole cell lysates were used as an input control. (**C**) The protein levels of FoxO1 and SYVN1 were detected by western blot. (**D**) The exogenous expression of FoxO1 was detected by western blot using Myc-tagged antibody. (**E-F**) The protein stability of FoxO1 was monitored by western blot in the presence of CHX. (**G**) S beads-enriched complex was detected by western blot. S beads, streptavidin beads; WCL, whole cell lysates. *, *P* < 0.05, **, *P* < 0.01, ***, *P* < 0.001

Ubibrowser analysis predicted that SYVN1 could mediate FoxO1 degradation as an E3 ubiquitin ligase. A series of mechanistic experiments were carried out to validate the direct association between SYVN1 and FoxO1. pMH-SFB-SYVN1 or pMH-SFB-FoxO1 were transfected into 293T cells. As shown in Fig. 5A, overexpression of SYVN1 or FoxO1 was confirmed by western blot by using the Flag antibody. Streptavidin beads successfully enriched SFB-SYVN1 and FoxO1, and vice versa (Fig. 5A). FoxO1 was immunoprecipitated by an anti-SYVN1 antibody in Hep3B and MHCC97H cells, thereby validating the SYVN1/FoxO1 interaction through Co-IP (Fig. 5B). Additionally, FoxO1 was upregulated in HCC cells whereas SYVN1 expression was downregulated (Fig. 5C). In addition, knockdown of SYVN1 decreased SYVN1 expression, resulting in the upregulation of FoxO1 in HCC cells (Fig. 5C). To further check whether SYVN1 regulated FoxO1 expression at the post-translational level, Myc-tagged FoxO1 was overexpressed in 293T cells. The proteasome inhibitor MG132 significantly increased FoxO1 level (Fig. 5D), indicating that ubiquitin-proteasome pathway might be involved in FoxO1 degradation. MG132 also inhibited FoxO1 degradation in the presence of the protein synthesis inhibitor CHX, compared with the corresponding control (Fig. 5E). Intriguingly, silencing of SYVN1 exerted a similar effect on FoxO1 protein stability in Hep3B and MHCC97H cells, in which the degradation rate of FoxO1 was slowed down by sh-SYVN1 (Fig. 5F). The ubiquitination of FoxO1 was further confirmed by Co-IP. In 293T cells that exogenously expressed Myc-tagged FoxO1, SFB-tagged SYVN1, and HA-tagged Ubiquitin, streptavidin beads significantly enriched HA-tagged Ubiquitin (Fig. 5G), indicating that SYVN1 played an indispensable role in the ubiquitination and degradation of FoxO1.

### SYVN1 modulates FoxO1 to promote immune escape, cell growth, and metastasis

**Fig. 6:**
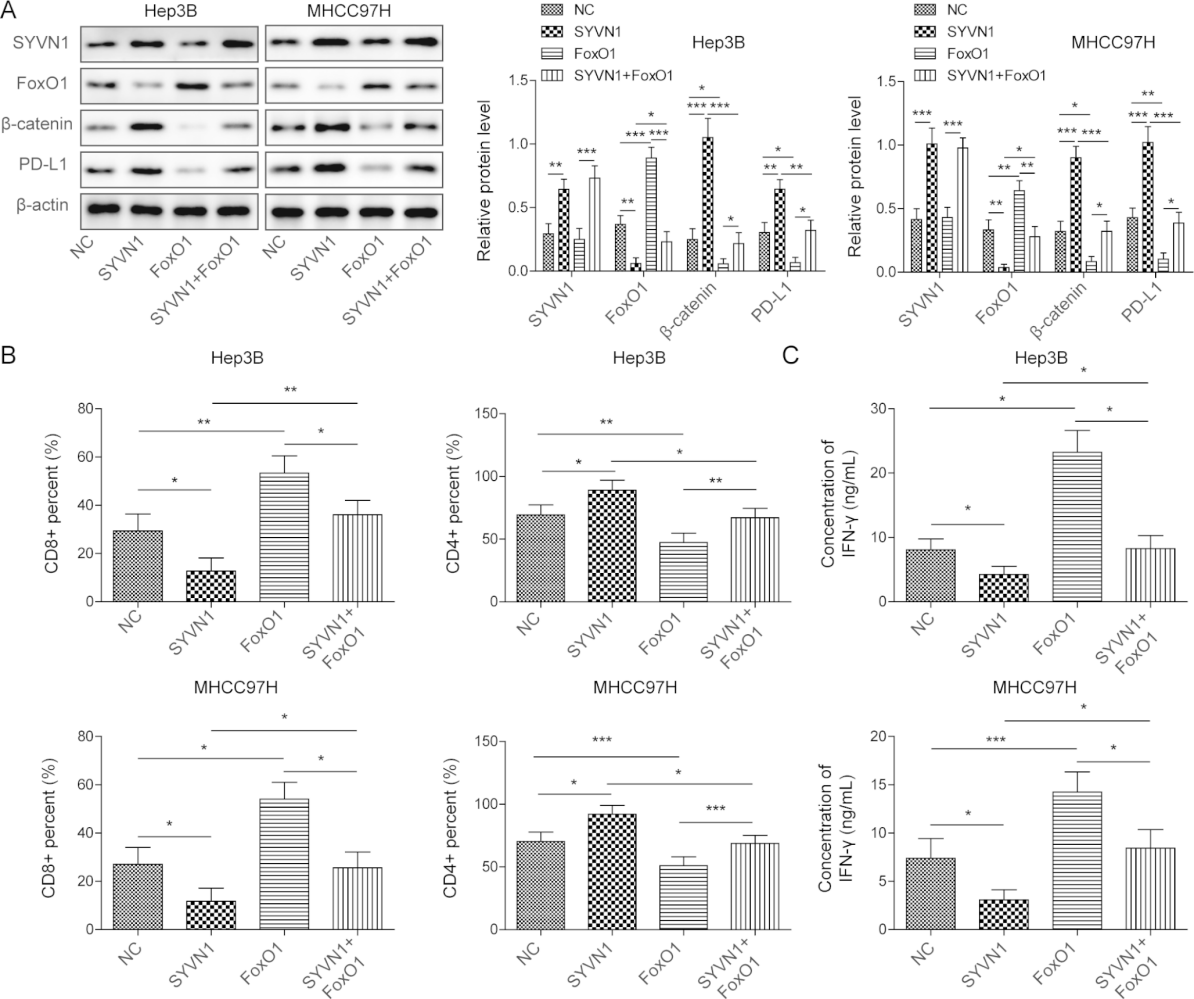
SYVN1 promotes immune escape via modulating FoxO1. (**A**) The protein levels of SYVN1, FoxO1, β-catenin and PD-L1 were detected by western blot. (**B**) The proportion of CD3+/CD8 + or CD3+/CD4 + T cells were detected by flow cytometry. (**C**) The secreted IFN-γ level was assessed by ELISA assay. *, *P* < 0.05, **, *P* < 0.01, ***, *P* < 0.001

To further unravel the function of the SYVN1/FoxO1 axis in HCC cells, overexpression experiments were conducted in both Hep3B and MHCC97H cells. As anticipated, transfection of a SYVN1 or FoxO1 overexpression construct successfully induced the expression of SYVN1 or FoxO1 in HCC cells, respectively. SYVN1 overexpression downregulated FoxO1 while upregulating β-catenin and PD-L1 expression. By contrast, FoxO1 overexpression had no significant effect on SYVN1 level while reversing SYVN1-induced β-catenin and PD-L1 levels in Hep3B and MHCC97H cells **(**Fig. 6A). In the HCC and PBMCs co-culture model, SYVN1 overexpression decreased the percentage of CD3+/CD8 + T cells, while FoxO1 overexpression led to a rebound in CD8 + T cell count. Consistently, the SYVN1-increased CD3+/CD4 + T cell population was abolished by FoxO1 overexpression (Fig. 6B). An ELISA assay further showed that SYVN1 significantly inhibited IFN-γ secretion, whereas FoxO1overexpression partially reversed this effect in both HCC cells (Fig. 6C). In accordance with the results of knockdown studies, SYVN1 overexpression promoted cell growth and colony formation in Hep3B and MHCC97H cells, and FoxO1 overexpression attenuated these positive effects of SYVN1 (Supplementary Fig. 2A-B). SYVN1-enhanced metastatic properties of HCC cells were abrogated by FoxO1 overexpression (Supplementary Fig. 2C). Functional studies indicate that FoxO1 serves as an important downstream effector of SYVN1 in HCC cells.

### SYVN1 silencing inhibits immune escape of HCC cells in vivo, possibly through the FoxO1/PD-L1 axis

**Fig. 7:**
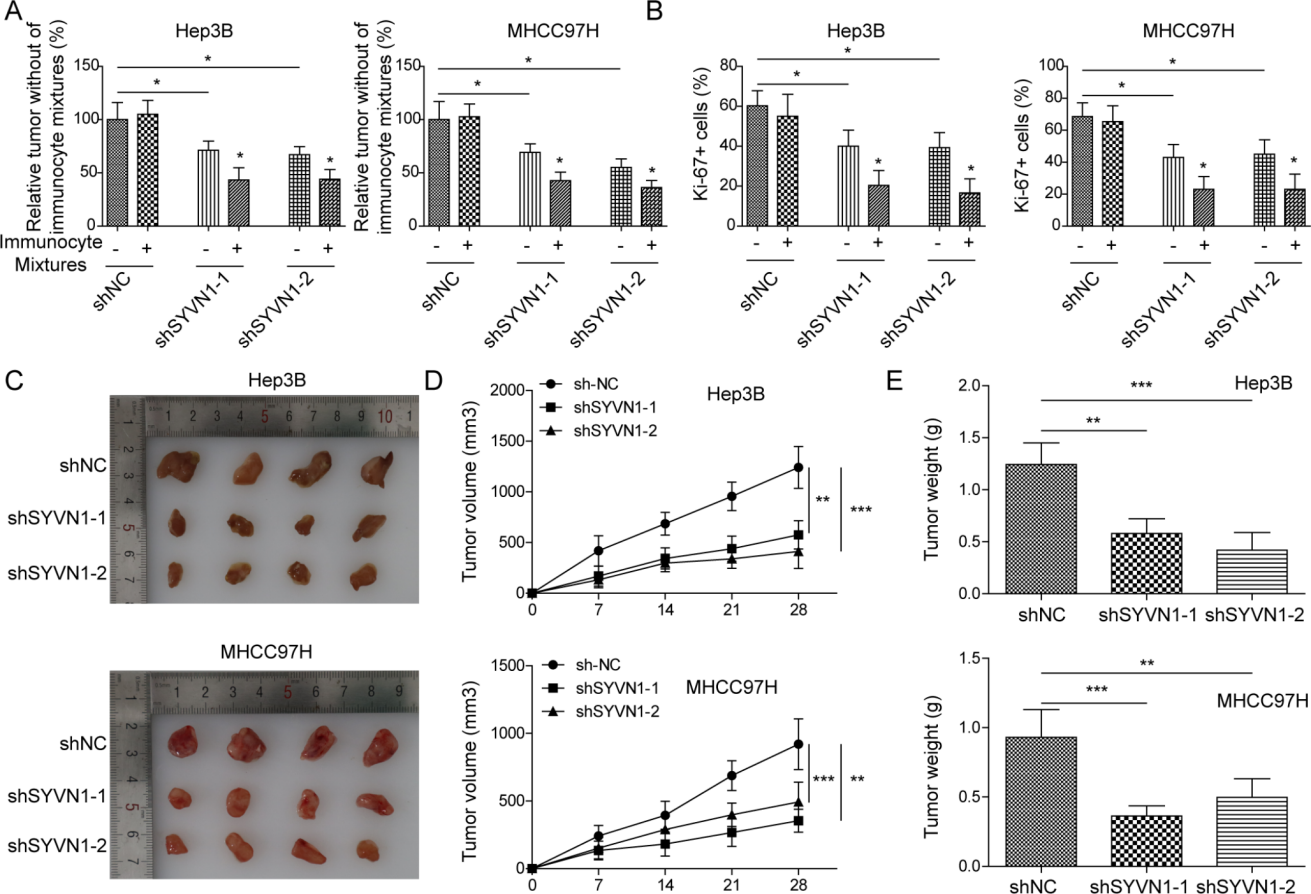
Silencing of SYVN1 inhibits immune escape of HCC cells, possible via FoxO1/PD-L1 axis in vivo. NOD/SCID mice were subcutaneously injected with stably transfected HCC cells to establish xenograft model. (**A**) Tumor regression rate was calculated. (**B**) Percentage of Ki67 + cells in tumor sections. (**C**) Photographs of xenograft tumors. (**D**) Quantitative analysis of tumor volume. (**E**) Quantitative analysis of tumor weight. Data are presented as mean ± SD. *, *P* < 0.05, **, *P* < 0.01, ***, *P* < 0.001

To further validate these findings in vivo, a xenograft model was established. Mice were administered with transfected HCC cells in the presence or absence of immunocyte mixtures. H&E staining revealed that knockdown of SYVN1 or immunocyte mixtures alleviated the histopathological features of HCC, and the improvement was more prominent in the sh-SYVN1 + immunocyte mixtures group (Supplementary Fig. 3). SYVN1 down-regulation or PBMC treatment reduced PD-L1 and Ki-67 expression, which was more pronounced in the SYVN1 silencing combined with PBMC treatment groups. After SYVN1 was depleted, SYVN1 expression decreased and FoxO1 expression increased in tumors, whereas PBMC administration had no effect on the expression of these proteins (Supplementary Fig. 3). In addition, sh-SYVN1 significantly reduced tumor regression rate, and the addition of immunocyte mixtures further decreased the tumor regression rate (Fig. 7A). Similarly, sh-SYVN1 or immunocyte mixtures reduced the percentage of Ki-67^+^ cells; however, the proportion of Ki-67^+^ cells was much lower in sh-SYVN1 + immunocyte mixture groups (Fig. 7B), suggesting that sh-SYVN1 or/and immunocyte mixtures decreased tumor cell proliferation. We next evaluated the effects of SYVN1 on tumor growth in vivo. As presented in Fig. 7C-E, the xenograft tumors derived from SYVN1-knockdown HCC cells exhibited smaller tumor size, volume and weight, compared with control group. Together, these findings suggest that SYVN1 silencing inhibits immune escape of HCC cells in vivo, possibly via the FoxO1/PD-L1 axis.

## Discussion

HCC is considered an immunogenic malignancy [28, 29]. Emerging evidence has illustrated that dysregulation of the immune system contributes to HCC development and progression, including changes in immune cells, cytokines, chemokines, immune ligands, and receptors [29]. Moreover, the majority of HCC patients do not benefit from ICIs, although ICIs provide improved clinical outcomes for the responders [2]. There is a pressing need to identify the biomarker of ICIs response. In the current study, we found that SYVN1 silencing or FoxO1 overexpression decreased PD-L1 expression, and inhibited immune escape, cell proliferation, and metastasis in HCC cells. FoxO1 regulated the level of PD-L1 either directly or indirectly through β-catenin. Moreover, SYVN1 acted as an E3 ubiquitin ligase for FoxO1, thereby promoting immune escape, cell proliferation, and metastasis. Our findings underscore potential implications for HCC immunotherapy.

TCGA and clinical data showed that SYVN1 was elevated in HCC tissues, which was consistent with previous findings [18]. Based on the data in starBase, 374 HCC tissues and 50 normal tissues were analyzed. SYVN1 was increased by 1.51-fold in HCC tissues, and lower level of FoxO1 (0.47-fold) was found in HCC tissues, compared with that in normal tissues. Besides our clinical data with relatively small sample size, starBase data also demonstrated the upregulation of SYVN1 and downregulation of FoxO1 in HCC tissues.

In HCC cell lines, HuH7 and LM3 cells, SYVN1silencing greatly suppresses cell growth, migration, and angiogenesis, and it also inhibits tumor growth in vivo [18]. In this study, similar results were consistently observed in Hep3B and MHCC97H cells. In addition to the metastatic functions of SYVN1, we showed that SYVN1 also played a crucial role in regulating immune evasion. SYVN1 depletion upregulated the proportion of cytotoxic CD8 + T cells, which further potentiated the anti-tumor effects of CD8 + T cells. Moreover, autocrine and paracrine IFN-γ enhances cytotoxicity of CD8 + T cells [30]. In HCC cell and PBMC co-culture model, sh-SYVN1-mediated IFN-γ induction exacerbated the cytotoxic effects of CD8 + T cells, inhibiting immune escape. Furthermore, several studies have demonstrated the tumor suppressive role of FoxO1 in HCC [31]. For instance, FoxO1 overexpression reverses the effect of miR-196a and inhibits cell proliferation in HCC [32]. In contrast, FoxO1silencing promotes HCC cell growth, colony formation, and migration [33]. In accordance with these reports, we confirmed the anti-tumor effects of FoxO1 in HCC cells. Additionally, FoxO1 overexpression also suppressed immune evasion. It is worth noting that SYVN1 positively regulated PD-L1 expression, while FoxO1 negatively modulated PD-L1 level. SYVN1 knockdown/FoxO1 overexpression and anti-PD-L1 antibody exerted a similar effect on CD4+/CD8 + ratio and IFN-γ secretion, indicating that PD-L1 may play a critical role in SYVN1 knockdown- or FoxO1 overexpression-regulated immune evasion in HCC.

FoxO1 has been identified as a transcriptional activator of PD-1 that contributes to CD8 + T cell survival during chronic viral infection [34]. In this study, we demonstrated that FoxO1 served as a transcription repressor of PD-L1, directly binding to PD-L1 promoter and suppressing its promoter activity. Beside this regulatory mechanism, an alternative β-catenin-dependent mechanism was also identified in HCC cells. FoxO1 reduced β-catenin expression, and the lack of β-catenin further resulted in reduced PD-L1promoter activity. Our findings were consistent with previous reports that FoxO1 restrains β-catenin nuclear translocation in HCC cells, suppressing downstream target gene expression [20]. In glioblastoma, depletion of β-catenin suppresses PD-L1 expression and facilitates activation and tumor infiltration of CD8 + T cells, thus inhibiting immune evasion [21]. In accordance with this study, functional experiments showed that SYVN1/FoxO1-mediated upregulation of β-catenin led to decrease of CD8 + T cells and IFN-γ secretion in HCC cell and PBMC co-culture model. In vivo, SYVN1 deficiency suppressed immune escape and lung metastasis, possibly via the FoxO1/β-catenin/PD-L1 axis. The detailed mechanism merits further investigation using various mouse models in future research.

A mechanistic study has shown that SYVN1 directly binds to Hsp90 and enhances the ubiquitination of EEF2K in HCC cells [18]. Intriguingly, FoxO1 was identified as a novel substrate of the ubiquitin ligase SYVN1 in Hep3B and MHCC97H cells. Co-IP confirmed the direct interaction between SYVN1 and FoxO1, as well as SYVN1-mediated FoxO1 ubiquitination. These findings indicate that SYVN1 promotes FoxO1 turnover via ubiquitin-proteasome pathway. SYVN1-induced immune evasion, cell growth, migration and invasion were reversed by FoxO1 overexpression, indicating that FoxO1 acts as a critical downstream effector of SYVN1 in HCC. In vivo findings revealed that FoxO1 was increased in xenograft tumors derived from SYVN1-knockdown cells. In the future, rescue studies are necessary to determine the role of FoxO1 in vivo.

**Fig. 8:**
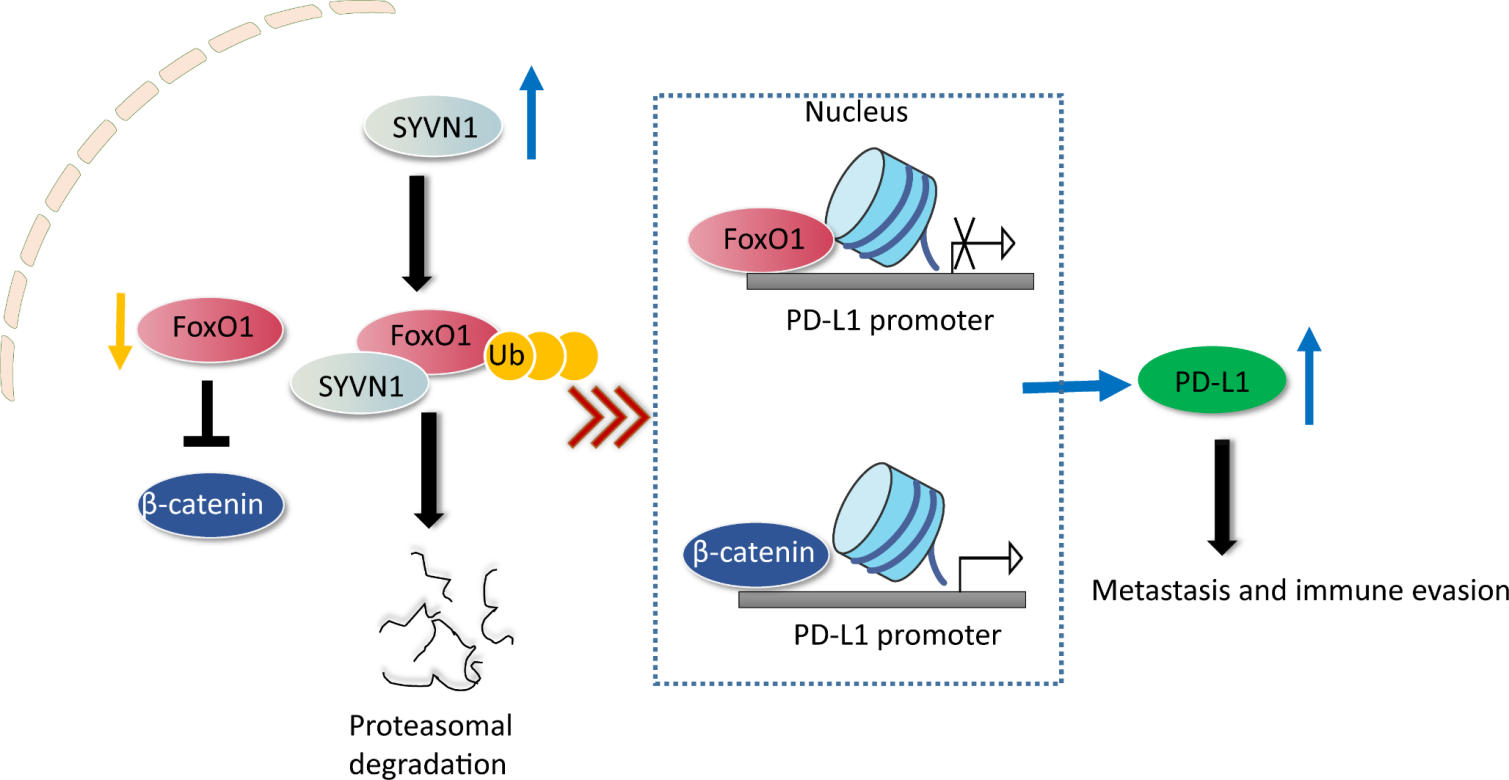
The working mechanism of the article. SYVN1 mediated the ubiquitin-proteasomal degradation of FoxO1, thus inducing β-catenin nuclear translocation, PD-L1-mediated metastasis and immune evasion of HCC.

In conclusion, SYVN1 downregulated FoxO1 expression via the ubiquitin-proteasome pathway. FoxO1 regulated PD-L1 level in a β-catenin-dependent or independent manner, contributing to PD-L1-mediated metastasis and immune evasion of HCC (Fig. 8). These findings provided novel insights into immune evasion in HCC and identified novel biomarkers of ICIs response.

### Electronic supplementary material

Below is the link to the electronic supplementary material.

**Supplementary materials**: 1?Figures and Figure legends. 2?Supplementary Tables and Table legends.

## Data Availability

All data generated or analysed during this study are included in this published article.

## Abbreviations

CCK-8: Cell Counting Kit-8
ChIP: Chromatin immunoprecipitation
CHX: Cycloheximide
Co-IP: Co-immunoprecipitation
FoxO1: Forkhead box protein O1
HBV: Hepatitis B virus
HCC: Hepatocellular carcinoma
HCV: Hepatitis C virus
H&E: Hematoxylin & eosin
ICIs: Immune checkpoint inhibitors
IF: Immunofluorescence
IHC: Immunohistochemistry
NASH: Non-alcoholic steatohepatitis
PBMCs: Peripheral blood mononuclear cells
PD-1: Programmed cell death 1
PD-L1: Programed cell death ligand 1
PFA: Paraformaldehyde
SYVN1: Synoviolin 1
TCGA: The Cancer Genome Atlas, TKIs, tyrosine kinase inhibitors
