# Supplementary material for: SYVN1 ubiquitinates FoxO1 to induce β-catenin nuclear translocation, PD-L1-mediated metastasis, and immune evasion of hepatocellular carcinoma
Source: Cell Oncol (Dordr). 2023 Apr 26;46(5):1285–99. doi: 10.1007/s13402-023-00811-y (PMC10618324; doi:10.1007/s13402-023-00811-y)
Supplement: Supplementary file 1 — Supplementary materials: 1?Figures and Figure legends. 2?Supplementary Tables and Table legends. [file 13402_2023_811_MOESM1_ESM.pdf]

Supplementary materials

1、Figures and Figure legends

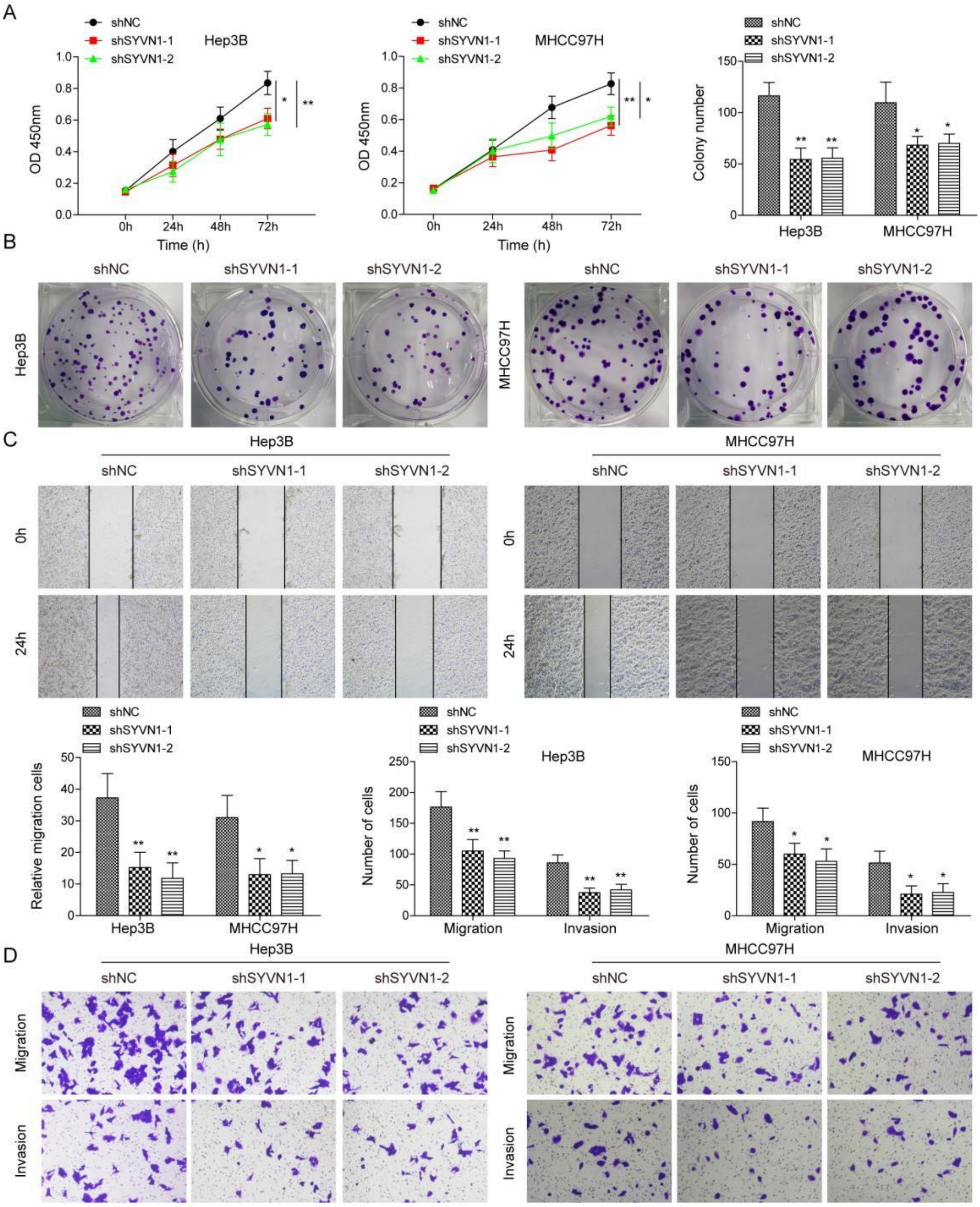

Supplementary Figure 1. Knockdown of SYVN1 inhibits cell proliferation and metastasis in HCC cells. (A) Cell proliferation was monitored by CCK-8 assay. (B) Colony forming ability was assessed by colony formation assay. (C-D) Cell migration and invasion were detected by Wound healing assay and Transwell assay, respectively.

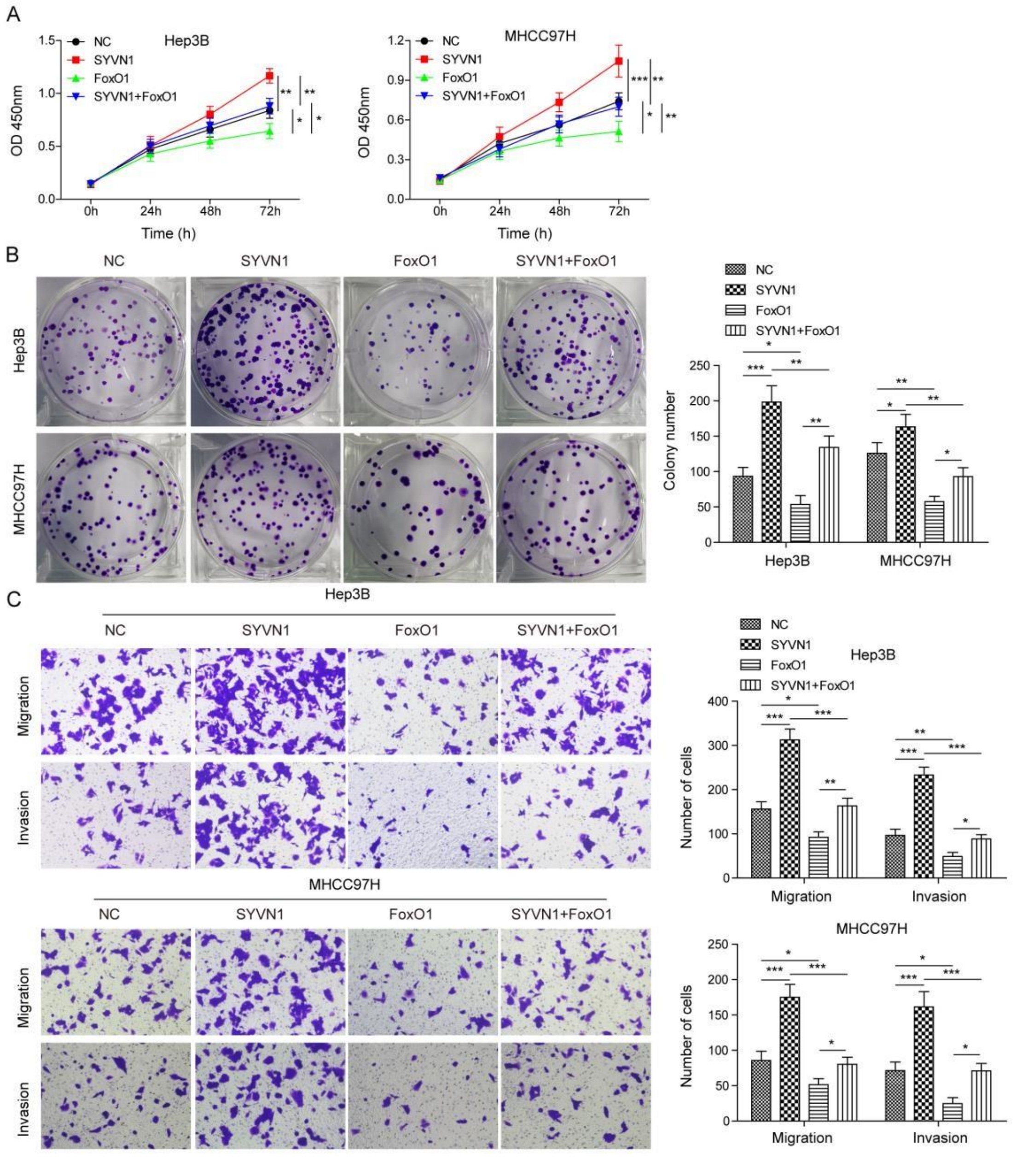

Supplementary Figure 2. SYVN1 promotes cell proliferation and metastasis via modulating FoxO1. (A) Cell proliferation was monitored by CCK-8 assay. (B) Colony forming ability was assessed by colony formation assay. (C) Cell migration and invasion were detected by Transwell assay. *, $P < 0.05$, **, $P < 0.01$, ***, $P < 0.001$.

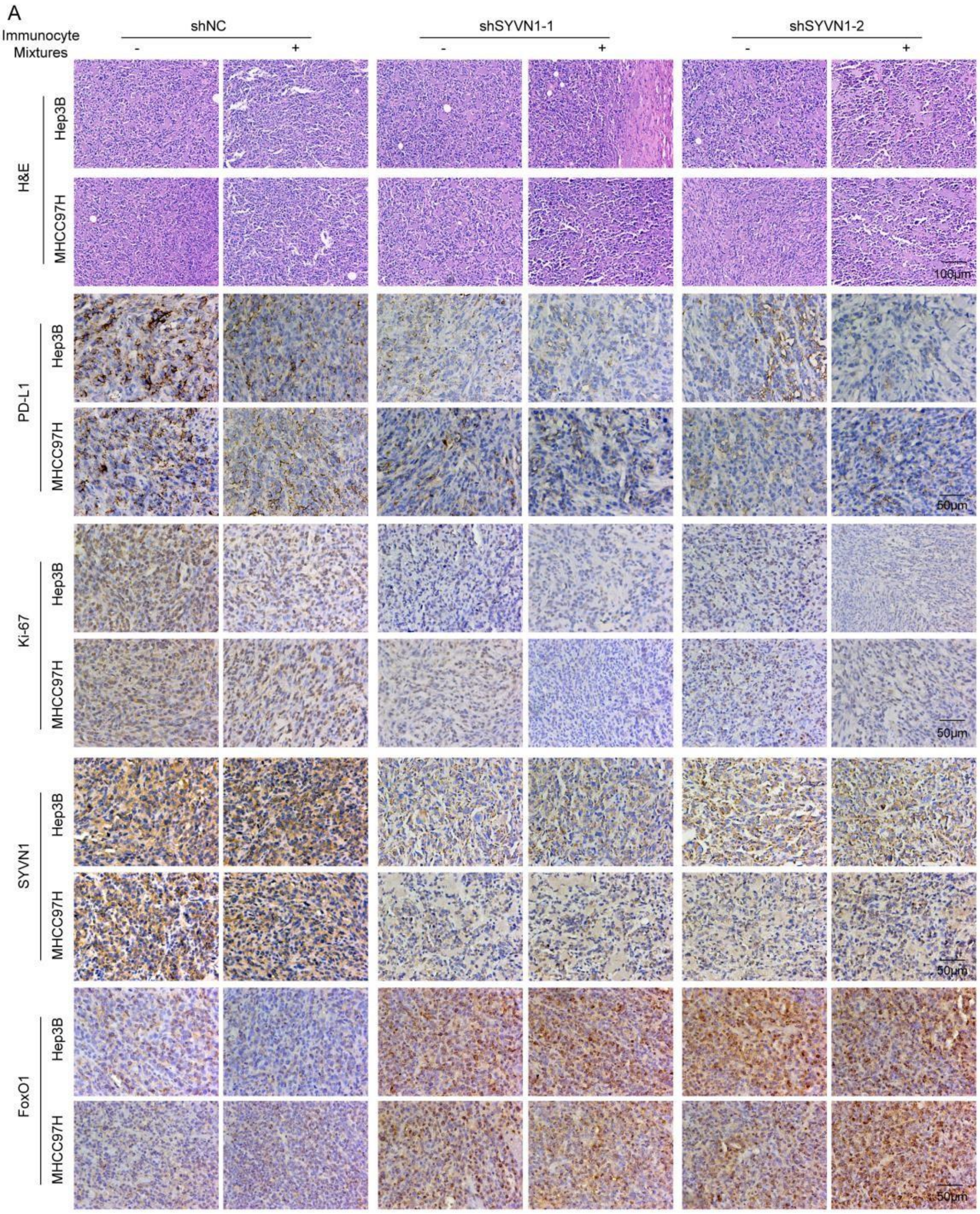

Supplementary Figure 3. Silencing of SYVN1 inhibits immune escape of HCC cells *in vivo*. NOD/SCID mice were subcutaneously injected with stably transfected HCC cells to establish xenograft model. (A) Representative images of H&E staining on tumor sections. Scar bar = 100 μm. Expression of PD-L1, Ki-67, SYVN1 and FoxO1 in tumors was detected by immunohistochemistry staining. Scar bar = 50 μm.

2、Supplementary Tables and Table legends

Supplementary Table 1 The primers used in the RT-qPCR.

**Primer**	**Sequence 5’-3’**
SYVN1 sense	CTTCGTCAGCCACGCTTATC
SYVN1 anti-sense	CCACGGAGTGCAGCACATAC
FoxO1 sense	CAGCAAATCAAGTTATGGAGGA
FoxO1 anti-sense	TATCATTGTGGGGAGGAGAGTC
GAPDH sense	CGCTCTCTGCTCCTCCTGTTC
GAPDH anti-sense	ATCCGTTGACTCCGACCTTCAC

Supplementary Table 2 Antibodies used in this study.

Antibody	Vendor	Catalog no.	Working dilution
SYVN1	Abcam	ab170901	WB (1:1000); IP (1 μg); IHC (1:100)
PD-L1	Abcam	ab205921	WB (1:1000); IF (1:200); IHC (1:200)
β-catenin	Abcam	ab32572	WB (1:2000); ChIP (1 μg)
FoxO1	Abcam	ab39670	WB (1:1000); ChIP (1 μg); IHC (1:100)
Myc	Abcam	ab32	WB (1:1000)
Flag	Abcam	ab205606	WB (1:2000)
HA	Abcam	ab9110	WB (1:2000)
Ki-67	Abcam	ab15580	IHC (1:200)
β-actin	Abcam	ab8226	WB (1:2000)

Supplementary Table 3 Correlation between SYVN1 and clinicopathological features in 30 HCC patients

Variable	SYVN1		p value
	Low	High	
**Age (years)**			0.7152
≤50	7	9	
> 50	8	6	
**Sex**			0.4621
Female	5	8	
Male	10	7	
**HBsAg**			0.6999
Negative	6	4	
Positive	9	11	
**Liver cirrhosis**			0.7104
No	8	10	
Yes	7	5	
**Serum AFP, ng/mL**			0.2635
≤20	11	7	
> 20	4	8	
**Serum ALT, U/L**			0.2451
≤75	8	12	
> 75	7	3	
**Tumor size (diameter, cm)**			**0.0253***
≤5	10	3	
> 5	5	12	
**NK cell proportion in blood**			**0.0209***
≤8%	6	13	
> 8%	9	2	
**Microvascular invasion**			**0.0142***
No	8	1	
Yes	7	14	
**Tumor number**			0.4497
Single	4	7	
Multiple	11	8	
**TNM**			0.4661
I/II	9	6	
III/IV	6	9	
